# Resilience Development Among Adult Patients With De Novo Acute Leukemia: A Qualitative Study

**DOI:** 10.1002/cam4.70343

**Published:** 2024-10-25

**Authors:** Danyang Li, Jing Li, Xinyu He, Meiyun Zhang

**Affiliations:** ^1^ Department of Nursing The First Affiliated Hospital Zhejiang University School of Medicine Zhejiang China

**Keywords:** acute leukemia, oncology, psychological resilience, psycho‐oncology, qualitative research

## Abstract

**Objective:**

This study aimed to explore the development process of psychological resilience among adult patients with de novo acute leukemia.

**Methods:**

This study utilized a descriptive qualitative approach, employing a purposeful sampling method to select a sample of 15 newly diagnosed patients with acute leukemia (AL) who underwent their initial induction chemotherapy treatment at the Hematology Department of the First Affiliated Hospital of Zhejiang University School of Medicine, China. Semi‐structured interviews were conducted with the selected patients. Content analysis methodology was used to analyze, summarize, and extract themes from the collected data.

**Results:**

Three categories emerged—namely, (1) negative period, (2) adaptive response phase, and (3) growth transformation period. The negative period occurs during the initial diagnosis and throughout the treatment cycle. However, influenced by both internal and external protective factors, including personal characteristics and social support, individuals enhance their psychological resilience through emotional regulation, mental adjustment, and adaptive strategies vis‐à‐vis healthcare decision‐making and disease management. Overall, psychological resilience development follows an upward spiral trajectory.

**Conclusions:**

This study identified that negative emotions and symptom clusters impede the development of patients' psychological resilience. Moreover, it revealed a substantial need for disease‐related information among patients. Therefore, healthcare professionals should prioritize addressing the negative emotions of patients, early identification of protective factors, dynamic monitoring of symptom clusters, effective management, and provision of psychological counseling and interventions. Simultaneously, providing personalized, professional, and systematic disease‐related information is vital for promoting psychological resilience development.

## 1 Introduction

Acute leukemia (AL) is a malignant clonal disease originating from hematopoietic stem cells that has an acute onset, rapid progression, severe symptoms, and a low survival rate [1]. Thus, it has become a major public health issue of global concern. Statistics reveal that leukemia's incidence and mortality rates rank 13th and 10th among all cancers, respectively, and continue increasing annually [2, 3]. The most common type of leukemia is AL, which is marked by the excessive proliferation of abnormal blast and immature cells in the bone marrow, and frequently accompanied by fever, bleeding, infection, and anemia. Untimely treatment may pose life‐threatening risks. Notably, chemotherapy is the most effective treatment method [4]. Specifically, induction chemotherapy is the frontline option for patients with de novo AL. However, patients undergoing induction chemotherapy frequently experience varying degrees and symptoms of physical discomfort and are at a high risk of life‐threatening complications [5]. Patients with AL typically experience higher levels of negative emotions, including anxiety and depression, than those undergoing noninitial treatment [6, 7, 8]. Severe anxiety and depression can worsen the patient's condition and even trigger suicidal thoughts [9, 10]. The “Healthy China 2030 Planning Outline” emphasizes enhancing early detection and timely intervention for psychological issues in key populations. Urgent attention and management are crucial for addressing the psychological challenges of patients with de novo AL.

Psychological resilience is key in how patients cope with challenging diseases, such as AL. The American Psychological Association defines resilience as the process of positive adaptation to adversity, tragedy, threats, trauma, or other significant sources of stress [11]. It is a dynamic process developing continuously in response to external changes [12]. Considering the severe physical and psychological stressors faced by patients with AL, understanding psychological resilience development in this population is crucial.

Current research on psychological resilience among adult patients with AL, particularly those with de novo AL, is limited. However, the literature has identified resilience's importance in helping leukemia patients cope with their disease [13]. Domestic research has indicated that factors such as personality traits, social support, emotional states, and economic conditions significantly influence resilience. For example, personality, educational level, and economic status impact resilience among older adults with acute myeloid leukemia [14]. Furthermore, lower resilience is associated with higher anxiety and depression levels [15], and resilience is also closely linked with quality of life. Evidence suggests that resilience positively correlates with quality of life and reduces anxiety and depression [16]. Additionally, interventions such as Promoting Resilience in Stress Management have proven effective in enhancing resilience, thereby improving cancer‐specific quality of life and psychological well‐being among leukemia patients [17]. Despite these findings, qualitative research exploring the dynamic development of resilience over the disease's course, particularly in the cultural context of Chinese patients, is lacking. Considering AL's unique nature, patients may experience significant impacts on resilience development from the onset of symptoms through various treatment stages. However, the dynamic changes in resilience at each stage remain unclear. Owing to the complex interplay of the disease, psychological factors, family, society, and the healthcare system, whether findings from other diseases can be applied to adult patients with de novo AL in China is uncertain.

Previous qualitative research on psychological resilience has explored diverse patient groups, such as those with burns, HIV, and cancers. For example, phenomenological research on liver cancer patients undergoing treatment has identified resilience themes such as crisis and adaptation [18]. Meanwhile, grounded theory analysis of burn patients' recovery highlighted resilience development stages [19]. Studies on lung cancer and HIV patients have underscored the role of protective factors, such as hope, self‐efficacy, and social support, in resilience development [20, 21]. However, considering the unique challenges faced by adult patients with de novo AL, research specifically examining the dynamic development of resilience in this population, particularly within the Chinese cultural context, is needed. Guided by Richardson's psychological resilience development process model [22], this descriptive qualitative study explores psychological resilience development among adult patients with de novo AL during initial treatment within the Chinese cultural context, aiming to inform targeted intervention strategies that healthcare professionals can utilize.

## 2 Methods

### 2.1 Design

This study utilized a descriptive qualitative approach; for data collection, face‐to‐face semistructured interviews were conducted. The Medicine Ethics Committee of the First Affiliated Hospital of Zhejiang University School approved this study(Ref.: 2022/748). All participants provided written informed consent. This study's reporting followed the Consolidated Criteria for Reporting Qualitative Studies (COREQ) [23].

### 2.2 Theoretical Framework

This study used Richardson's psychological resilience model [22] to structure the research design and analyze the collected data. Richardson's model elucidates the developmental process of psychological resilience [22] based on psychological, spiritual, and biological equilibrium models; thereby, psychological resilience's manifestations are classified into regressive reorganization (returning to preadversity levels) and resilient reorganization or restructuring, suggesting that multiple dissociations and restructurings may occur during the same period.

### 2.3 Participants

The participants were selected using purposive sampling combined with maximum variation (in terms of age, educational level, residence, personality, type of medical insurance, occupation, family income, and comorbidities). The inclusion criteria were as follows: (a) aged 18–60 years; (b) diagnosed with AL by bone marrow aspiration, with blood neutrophils ≥ 1–2 × 10^9^/L and platelets ≥ 30 × 10^9^/L after recovery from the first induction chemotherapy; (c) aware of the diagnosis and willing to cooperate with the interviews; (d) clear thinking and adequate communication skills. These criteria were selected for the interview for the following reasons: Adults with de novo AL undergoing initial chemotherapy exhibit significant bone marrow suppression on days 10–14, followed by recovery; neutrophils at 1–2 × 10^9^/L, platelets at 30 × 10^9^/L, and the absence of infections render patients eligible for discharge; and at this stage, the patient's condition is relatively stable.

### 2.4 Data Collection

The research team reviewed relevant literature, and based on Richardson's psychological resilience development model, an interview outline was finalized after pretesting with three patients using the following questions: May you tell us about your diagnosis and how you felt at the time? How did you cope when you found out you had the disease? What help did you receive? How has your daily life changed since your diagnosis? What were your experiences and feelings during the treatment process (specifically, during diagnosis, induction chemotherapy, myelosuppression, and recovery after chemotherapy)? How do you view and cope with the hardships and ups and downs of the treatment process? What was the strength that kept you going during this period? How do you rate your performance during this time? How do you feel about your current state of life? How do you envision your life in the future, and do you have any plans? Is there anything else you want to tell me?

All interviews were conducted by LDY, who has qualitative research experience and more than 15 years of clinical experience in hematology‐oncology nursing. Before conducting the prearranged interviews in a consultation room, we introduced the purpose and procedures to the participants. Each interview lasted 25–53 min; theoretical saturation was reached after 15 patients. The total duration of all interviews was 560 min. Data were collected from November 2022 to March 2023.

### 2.5 Data Analysis

Conventional content analysis was used to synchronize data collection and analysis. Interview notes and a reflection diary were integrated to conduct open coding and facilitate a holistic understanding of the textual data. Similar or related codes were organized into themes and subthemes; themes, subthemes, and codes were clearly defined; and the ultimate themes and subthemes were determined by elucidating intersubject connections.

Two researchers (LDY and LJ) acquired, analyzed, summarized, and supplemented the recording materials and field notes within 24 h. Three researchers (LDY, LJ, and HXY) coded the data. Notably, LJ and HXY are both nursing postgraduates with qualitative research experience. All the themes were reviewed by all research group members.

## 3 Results

Among the 15 participants, nine were female (60%) and six were male (40%); their ages ranged from 23 to 60 years, with a mean age of 39.2 years (SD = 11.16). Regarding marital status, three and 12 participants were unmarried and married, respectively. Regarding the self‐reported personality traits from the Myers‐Briggs Type Indicator (MBTI), five participants were introverts, four were ambiverts, and six were extroverts. Regarding educational level, 1, 3, 3, 2, 5, and 1 participants had elementary education, middle school education, a high school or vocational school diploma, a specialized school diploma, a bachelor's degree, and a master's degree, respectively. Concerning geography, 5, 1, 7, and 2 participants lived in rural areas, towns, county, and provincial capitals, respectively. Regarding employment status, 12, 1, and 2 participants were employed, retired, and inactive, respectively. Table 1 details the participants' demographics.

**TABLE 1:** Demographic characteristics of the study participants (*n* = 15).

No.	Age (year)	Gender	Marital status	Personality	Domicile	Education	Occupation	Monthly family income	Number of children	Faith	Health insurance type	Complications
1	43	Male	Married	Introvert	County	Bachelor's degree	Public servant	> 5000	1	N/A	Public medical care	N/A
2	46	Female	Married	Extrovert	Rural area	Elementary education	N/A	> 10,000	2	N/A	Urban medical insurance	N/A
3	30	Male	Married	Extrovert	County	Middle school education	Freelancer	> 10,000	1	N/A	Urban medical insurance	N/A
4	38	Male	Married	Extrovert	County	Bachelor's degree	Public servant	> 10,000	1	N/A	Public medical care	N/A
5	60	Female	Married	Extrovert	Provincial capital	High school diploma	Retired	> 10,000	1	N/A	Urban medical insurance	Y
6	38	Female	Married	Ambivert	County	Middle school education	Freelancer	> 10,000	2	N/A	Urban medical insurance	N/A
7	23	Female	Unmarried	Extrovert	Rural area	Bachelor's degree	Office employees	> 5000	0	N/A	Urban medical insurance	N/A
8	27	Female	Unmarried	Ambivert	Rural area	Master's degree	Office employees	> 10,000	0	N/A	Urban medical insurance	N/A
9	45	Female	Married	Extrovert	Provincial capital	Bachelor's degree	Office employees	> 10,000	2	N/A	Public medical care + Commercial insurance	N/A
10	32	Male	Married	Ambivert	County	Middle school education	Freelancer	> 10,000	2	Buddhism	New Rural cooperative medical	N/A
11	48	Male	Married	Introvert	Rural area	Vocational school diploma	Freelancer	> 10,000	1	N/A	Urban medical insurance	N/A
12	31	Male	Married	Introvert	County	Specialized school diploma	Office employees	> 10,000	0	N/A	Urban medical insurance	Y
13	44	Female	Married	Ambivert	Town	Bachelor's degree	Teacher	> 10,000	1	N/A	Public medical care	N/A
14	59	Female	Married	Introvert	Rural area	High school diploma	Retired	> 10,000	1	N/A	Urban medical insurance	N/A
15	24	Female	Unmarried	Introvert	County	Specialized school diploma	N/A	> 10,000	0	N/A	Public medical care	N/A

### 3.1 Categories and Subcategories

Conventional content analysis was employed for data analysis; consequently, three categories with nine subcategories were identified (Table 2).

**TABLE 2:** Categories and subcategories.

Categories	Subcategories
Negative period	Negative emotions during diagnosis Negative emotions during treatment
Adaptive response phase	Internal protective factors: personal traits External protective factors: social support systems Emotional regulation: self‐expression and self‐adjustment Mental regulation: hope, establishing life objectives, and constructing survival motivation Adaptive strategies vis‐à‐vis healthcare decision‐making and disease management
Growth and transformation period	Positive outcomes Unexpected gains

#### 3.1.1 Negative Period

##### 3.1.1.1 Negative Emotions During Diagnosis

The AL diagnosis induced a spectrum of negative emotions—including shock, fear, devastation, dread, self‐denial, anxiety, and depression—among the participants. It represented a significant stressor necessitating the patient to adapt to a new reality. During the diagnosis period, the patient's ignorance, worries, and fears concerning the disease; uncertainty regarding the future; and the disease's impact on life, work, family, and the individual all contributed to precipitating negative emotions.

“*It hit me out of the blue*, *being a healthy ex‐serviceman*. *This illness felt like a real punch in the gut*, *something I never saw coming*” (P4).

“*Juggling this sickness and taking care of my kids and elderly parents at the same time is really tough*. *I'm afraid to tell my parents about my situation; I'm worried it might upset them or make them sick too* (*voice trailing off*)” (P11).

“*After being told I had leukemia*, *it hit me with a lot of fear*, *like I was just waiting around to die*.” (P12).

##### 3.1.1.2 Negative Emotions During Treatment

Adult patients with de novo acute leukemia may experience recurring negative emotions because of a lack of knowledge regarding disease treatment, symptoms during treatment, disease progression, the resultant financial burden, and misinformation.

“*I was afraid that I would die within a few days of beginning chemo*.” (P1).

“*I was really scared when other patients told me how tough the first chemo would be*. *But when I went through it*, *it wasn't as bad as I thought*. *The worst part was probably when I got a lung infection and COVID‐19 on top of the chemo's neutropenic phase*. *I experienced recurring fevers and felt extremely weak and disoriented*. *There were moments when I contemplated giving up*” (*P6*).

“*The doctor suggested a costly new medication for the next round of therapy*. *Its efficacy for me is still uncertain*. *I felt devastated and helpless*.” (P8).

“*I was scared about chemotherapy*. *I was also feared that I would die suddenly from intracranial hemorrhage like the patient in the adjacent bed*.” (*P12*).

“*I didn't know what to expect from chemotherapy*, *and I was really worried*. *By the third day*, *I had a terrible time with my stomach*, *throwing up after I ate and feeling super tired and down all the time*.” (P13).

“*On the second day of chemotherapy*, *I began to experience vomiting*, *which occurred 3 to 4 times per day*. *Additionally*, *I developed oral ulcers*, *which*, *due to my heightened sensitivity to pain*, *made it difficult for me to eat*. *During the chemotherapy period*, *I also faced issues with bowel movements*, *requiring the use of laxatives*, *which was quite distressing*. *When my platelet count was low*, *my menstrual cycle coincided*, *resulting in a heavy flow and significant blood loss*, *which left me feeling extremely weak*. *That night*, *I had a moment of feeling as if I could not endure the situation any longer*.” (P15).

#### 3.1.2 Adaptive Response Phase

In Richardson's model of the developmental process of psychological resilience, although the individual initially experiences a balanced biopsychospiritual homeostasis, stressors begin interacting with personal protective factors and result in system breakdown and reintegration.

##### 3.1.2.1 Internal Protective Factors: Personal Traits

Personal traits—such as optimistic attitudes, strong belief and fortitude, and the ability to think independently—motivate patients to perceive and cope with posttraumatic events and promote psychological resilience development. The interview responses revealed that self‐reported extroverts and ambiverts displayed more optimistic attitudes than introverts. However, not all extroverted patients exhibited these traits.

“*I believe that it is important to live with positivity*.” (P9).

Most interviewees have faced challenges in life, demonstrating strong adaptability and possessing firm beliefs and perseverance. “*To get through is to win*, *and I have always had this attitude when dealing with challenges*.” (P13).

Patients exhibit individual differences in their attitudes toward the disease, and independent and rational thinking positively contributes to their developing psychological resilience. “*I was really scared when I heard the diagnosis*, *but I'm ready to face whatever comes*, *no matter how tough it might be*.” (P9).

##### 3.1.2.2 External Protective Factors: Social Support Systems

Robust social support enhances patients' coping, information acquisition, and disease management. The interview responses indicated that patients received social support predominantly in the form of emotional, informational, behavioral, and financial support.

“*After I got diagnosed*, *my sister stepped in to look after the kids so my husband could concentrate on helping me through my treatment*. *Plus*, *they chipped in financially to back my medical bills*.” (P6).

“*While I was in the hospital*, *I got lots of calls from family and friends*. *The docs and nurses were really patient*, *explaining my health situation in a way that made sense to me*, *which helped me understand my condition better*.” (P13).

##### 3.1.2.3 Emotional Regulation: Self‐Expression and Self‐Adjustment

Self‐expression refers to an individual expressing their feelings and emotions in writing or verbally [24]. Self‐expression contributes to the development of patients' psychological resilience in terms of emotional release, establishing support systems, information acquisition, self‐identification and acceptance, and reflection and meaning construction. “*I'm strong in the eyes of outsiders*, *but when I am sad*, *I find a place to bawl by myself*.” (P8).

Participants' self‐adjustment strategies involve seeking psychological balance through both upward and downward comparisons, spiritual introspection, and selective *perception*. “*I feel that I am in better health than the other patients in this ward*.” (P9).

Spiritual introspection occurs through mental and physical rest, whereby participants eliminate distractions, cope with the treatment's side effects, and, thereby, reduce their discomfort. “*I slept all the time while I was in chemo*.” (P15).

##### 3.1.2.4 Mental Regulation: Hope, Establishing Life Objectives, and Constructing Survival Motivation

Hope is a human force that enables individuals to utilize their environmental resources to facilitate healthy development and achievement [25]. “*I hope to see my daughter graduate from college*, *secure a job*, *and get married*.” (P11).

Participants aimed to set new life goals, driven by survival instincts, while seeking support and striving for self‐realization. “*Currently prioritizing treatment*, *with plans for pursuing postgraduate studies and a return to normalcy post‐recovery*.” (P7).

Consistent with previous studies, participants highlighted family commitment's significant role in motivating them to survive [26]. “*I am anticipating the arrival of my child in five months*, *and thus*, *I must persevere*.” (P12).

##### 3.1.2.5 Adaptive Strategies Vis‐à‐Vis Healthcare Decision‐Making and Disease Management

As healthcare in China develops, the development of medical resources has gradually equalized. Most interviewees reported a preference for high‐quality healthcare resources, considering factors such as disease severity, medical insurance, finances, and other relevant aspects. “*When I fell ill*, *with my platelet count being really low and things being serious*, *I chose this hospital because they have better payment coverage and top‐notch medical equipment*.” (P4).

Some participants acknowledged their disease, approached it objectively, detached from negative emotions, and adapted gradually. “*This disease is treatable*, *and the outcomes of treatment vary among individuals*. *Hence*, *I will cooperate with the medical treatment*.” (P6).

Some participants mentioned employing behavior reshaping and attention redirecting as strategies for improved disease management. “*If I get better*, *I will not neglect myself like I used to*, *with my sleep patterns reversed day and night*.” (P3) “*Throughout my treatment*, *I kept a close eye on my meds and tracked my blood work*. *I also wrote in a journal every day and tried to keep busy with different things*.” (P9).

#### 3.1.3 Growth Transformation Period

##### 3.1.3.1 Positive Outcomes

Participants who recognized and cherished life's value and found joy in the present exhibited heightened psychological resilience. “*I've learned to value life more*, *let go of some of my worries*, *savor each day*, *and make it a point to spend quality time with my family*.” (P4).

##### 3.1.3.2 Unexpected Gains

Some participants revisited the disease treatment process, appreciating the benefits of family and friends' concern for them. “*Since I got sick*, *my husband's been even more caring and supportive than ever*.”(P6).

Participants found solace after their diagnoses, recognized life's impermanence, and reevaluated life's meaning. “*Realizing that life's short and unpredictable*, *I've started to prioritize my health and well‐being over climbing the career ladder*, *looking for ways to live better and healthier*.” (P4).

## 4 Discussion

This qualitative study examines psychological resilience development among adult patients with de novo AL within the Chinese cultural context. Our findings reveal that resilience development is a dynamic process, characterized by an overall upward spiral. Patients frequently experience negative emotions during diagnosis and initial treatment, which impede resilience development. Previous studies have demonstrated high psychological distress levels among patients undergoing induction chemotherapy [27], with newly diagnosed patients exhibiting elevated anxiety and depression levels [8]. Our study builds on these findings by mapping negative emotions' trajectory, which typically begins with shock, fear, and anxiety. As patients accept their diagnosis, receive social support, engage in self‐regulation, and adopt active coping strategies, these negative emotions tend to diminish, though they may resurface because of treatment‐related challenges. The psychosocial state of patients with AL is closely linked to their cognitive biases and distortions [28]. Cognitive behavioral therapy and virtual reality‐based meditation interventions have shown promise in managing patients' emotional fluctuations and improving their quality of life [29, 30]. Incorporating these interventions into routine care may enhance patients' resilience, fostering positive coping mechanisms and strengthening psychological adaptability among them.

Further, our study explores in detail symptom clusters during the initial treatment phases, including diagnosis, induction chemotherapy, bone marrow suppression, and postchemotherapy recovery. Previous studies by Yang et al. [31] and Zhang [32] primarily focused on the prevalence and severity of individual symptoms, particularly those related to psychological, gastrointestinal, and fatigue clusters; however, our study deeply examines patients' experiences, attitudes, and coping strategies, highlighting these clusters' significant impact on emotional well‐being and treatment adherence, which further hinders psychological resilience development. Additionally, Brant et al. [33] introduced a dynamic symptom management model; meanwhile, our study underscores the importance of personalized, multidisciplinary approaches incorporating digital health technologies to optimize symptom management. By addressing the unique challenges posed by symptom clusters, our study contributes to a more comprehensive understanding of care for patients with AL, advocating for targeted interventions to improve their quality of life and treatment outcomes.

When transitioning into the adaptive response phase, patients leverage both internal and external protective factors to enhance their resilience. This study refines these factors' understanding, identifying optimism, determination, and rational thinking—along with comprehensive social support from spouses, family, healthcare providers, and peers—as critical in fostering resilience. Previous studies have emphasized various sociodemographic factors' impact on resilience [34]. Meanwhile, our findings highlight the dynamic role of social support, emotional and psychological regulation, and proactive coping strategies. Participants' positive attitudes toward treatment, shaped by past experiences, significantly bolster their resilience. To promote patient resilience, these protective factors should be a key focus when designing care interventions.

Additionally, our findings align with previous research, such as that by Jie et al. [35], which reported a strong demand for health system information and psychological support among adult patients with AL. However, unlike quantitative studies that primarily assess unfulfilled needs through surveys, our qualitative approach provides a more nuanced understanding of how patients seek and process health information. Newly diagnosed patients typically experience confusion and uncertainty, relying on various sources—such as textbooks, the internet, and peer consultations—to fill knowledge gaps. These sources' reliability and effectiveness vary, and patients' needs evolve throughout the treatment process. This study contributes to the field by highlighting current health information sources' limitations and the dynamic nature of patients' informational needs. In the healthcare reform context, the integrated healthcare management model has extended into various nursing fields, promoting active collaboration among doctors, patients, and caregivers [36]. This model offers personalized, professional, and systematic disease and treatment information, helping establish accurate disease understanding. Tailored guidance can be offered by analyzing health information‐seeking behaviors [37]. Utilizing platforms such as WeChat, social media, and the Internet enables diverse information dissemination strategies—crucial for helping patients effectively manage their disease and enhance their psychological resilience.

### 4.1 Study Limitations

This study has limitations. The theoretical saturation, while applicable to the study outcomes, may evolve over time. Additionally, the study was conducted solely during the COVID‐19 outbreak in China, including only subjects from the investigator's hospital, limiting generalizability. The results may lack external validity, affecting their representativeness.

### 4.2 Clinical Implications

The findings guide healthcare professionals to early identify protective factors for psychological resilience, manage patients' negative emotions promptly, dynamically monitor symptom clusters for effective intervention, and provide personalized, professional, and systematic disease‐related information.

## 5 Conclusions

This study found that personal traits and social support serve as protective factors for—whereas negative emotions and symptom clusters impede—psychological resilience development. Individuals enhance their psychological resilience through emotional regulation and positive coping, driven by various internal and external protective factors. Moreover, this study revealed a substantial need for disease‐related information among patients.

## Author Contributions

**Danyang Li:** conceptualization (equal), data curation (equal), formal analysis (equal), investigation (equal), methodology (equal), project administration (equal), resources (equal), writing – original draft (equal), writing – review and editing (equal). **Jing Li:** conceptualization (supporting), formal analysis (supporting), investigation (equal), writing – review and editing (equal). **Xinyu He:** data curation (equal), formal analysis (equal), investigation (equal), methodology (equal). **Meiyun Zhang:** conceptualization (supporting), data curation (lead), methodology (lead), project administration (equal), project administration (lead), resources (lead), supervision (equal), supervision (lead), writing – review and editing (equal), writing – review and editing (supporting).

## Ethics Statement

This study was approved by the Institutional Review Board of the First Affiliated Hospital of Zhejiang University School (Ref: 2022/748).

## Conflicts of Interest

The authors declare no conflicts of interest.

## Data Availability

All data analyzed in this study are available on request from the corresponding author. Due to the protection of participants' privacy, the data are not publicly available.
